# Preparation of a Hydrogel Nanofiber Wound Dressing

**DOI:** 10.3390/nano11092178

**Published:** 2021-08-25

**Authors:** Radek Jirkovec, Alzbeta Samkova, Tomas Kalous, Jiri Chaloupek, Jiri Chvojka

**Affiliations:** 1 Department of Nonwovens and Nanofibrous Materials, Faculty of Textile Engineering, Technical University of Liberec, 461 17 Liberec, Czech Republic; tomas.kalous@tul.cz (T.K.); jiri.chaloupek@tul.cz (J.C.); jiri.chvojka@tul.cz (J.C.); 2 Department of Material Engineering, Faculty of Textile Engineering, Technical University of Liberec, 461 17 Liberec, Czech Republic; alzbeta.samkova@tul.cz

**Keywords:** hydrogel, nanofibers, skin cover, alginate, polyvinyl alcohol

## Abstract

The study addressed the production of a hydrogel nanofiber skin cover and included the fabrication of hydrogel nanofibers from a blend of polyvinyl alcohol and alginate. The resulting fibrous layer was then crosslinked with glutaraldehyde, and, after 4 h of crosslinking, although the gelling component, i.e., the alginate, crosslinked, the polyvinyl alcohol failed to do so. The experiment included the comparison of the strength and ductility of the layers before and after crosslinking. It was determined that the fibrous layer following crosslinking evinced enhanced mechanical properties, which acted to facilitate the handling of the material during its application. The subsequent testing procedure proved that the fibrous layer was not cytotoxic. The study further led to the production of a modified hydrogel nanofiber layer that combined polyvinyl alcohol with alginate and albumin. The investigation of the fibrous layers produced determined that following contact with water the polyvinyl alcohol dissolved leading to the release of the albumin accompanied by the swelling of the alginate and the formation of a hydrogel.

## 1. Introduction

Accidents lead to the occurrence of a range of skin injuries which, in general, can be divided into epithelializing, granulating, slough present and necrotic tissue wounds [1], all of which, following treatment, are covered with traditional wound dressings. However, following recent scientific developments, wounds can now also be covered with nanofibrous skin covers supplemented by permeable films, foams and even hydrogels [2,3,4,5,6]. The ideal wound dressing should provide a moist environment, promote connective tissue synthesis and, naturally, be both sterile and non-toxic [2].

Nanofiber skin covers are fabricated from, for example, gelatin [4], acetate [7], polyvinyl alcohol [8] and polycaprolactone [9] via the electrostatic spinning technique [10] that produces fine fibers with diameters of 50 to 1000 nm, either from a polymer solution or from a melt [11]. Electrospinning is performed by either the direct current (DC) or alternating current (AC) spinning methods [12]. DC spinning employs a DC source to form the fibers, which requires an electrically active collector, while the AC spinning approach employs a high-voltage AC source. Since the polarity changes during AC spinning, no electrically active collector is required [13]. A further difference between DC and AC spinning concerns the potential to produce bulky fiber layers via the AC spinning method [14].

Skin covers can be fabricated from hydrogels [6], which comprise hydrophilic three-dimensional crosslinked structures that are able to absorb large amounts of water [15,16]. In order to ensure their stability, they need to be both physically and chemically crosslinked [17]. Hydrogels are similar to the extracellular matrix in terms of their composition and mechanical properties. They are able to serve as a support material for cells during the tissue regeneration process and allow for the diffusion of nutrients, metabolites and growth factors [18,19]. Hydrogels can be fabricated from both natural materials, i.e., gelatin [20], alginate [21] or chitosan [22], and synthetic materials, i.e., polyvinyl alcohol (PVA) [23], polyethylene oxide [24] and polyvinyl pyrrolidone [25], and are used to cover burn and surgical wounds and pressure ulcers [26].

Such materials can also be combined [27] to form composite skin covers that consist of a nanofiber structure and a hydrogel via a two-phase production process. Firstly, each material is produced separately and subsequently combined to form the composite material. However, new studies try to combine fibrous elements with hydrogel elements to form hydrogel fibers. Hydrogel fibers can be prepared in several ways, including wet spinning [28], electrospinning [29] or 3D printing [30]. However, the disadvantages of hydrogel fibers are their poorer mechanical properties and their difficult handling during possible application or packaging [31].

This study aimed to develop a new material that combines a nanofiber structure and a hydrogel. This objective was achieved via the production of a fibrous layer from the electrostatic spinning of PVA with alginate followed by crosslinking with glutaraldehyde. The study led to the production of a material from which the PVA dissolved upon contact with water, accompanied by the swelling of the alginate to form an alginate hydrogel.

## 2. Materials and Methods

### 2.1. Materials

Gelatin obtained from pork skin (Sigma-Aldrich, Hamburg, Germany), alginate (Sigma-Aldrich, Hamburg, Germany) and PVA (Mw 85,000–124,000, hydrolysis 87–89%, Sigma-Aldrich, Hamburg, Germany) were used for the preparation of the hydrogel fibers. Distilled water was selected as the solvent system. Albumin (Sigma-Aldrich, Hamburg, Germany) was selected for the modification of the fibrous layers. A solution of 25% of glutaraldehyde (Penta Chemicals, Prague, Czech Republic) was then used for the crosslinking of the resulting fibers.

### 2.2. Preparation

Solutions of gelatin at concentrations of 10, 12, 14 and 16 wt% were prepared for electrostatic spinning followed by the preparation of alginate solutions at concentrations of 2, 4, 6 and 8 wt% and PVA solutions at concentrations of 8, 10 and 12 wt%. In order to ensure that the polymers used dissolved completely, the solutions were stirred for 24 h and the PVA solution was heated to a constant temperature of 80 °C. The solutions were then spun using a Nanospider NS LAB laboratory device fitted with a string electrode. The spinning took place on a Spunbond-type base material.

Since the fibrous layers are soluble when exposed to an aqueous medium, they were crosslinked with glutaraldehyde in the form of a 25% aqueous glutaraldehyde solution, which was poured into a Petri dish and then placed in a desiccator. A grid was placed over the Petri dish upon which the fiber layers were positioned. Thus, crosslinking was conducted via exposure to the glutaraldehyde vapor.

### 2.3. Scanning Electron Microscopy and Analysis

The prepared nanofiber layers were coated with a 10 nm layer of gold using a Quorum Q150R ES device (Quorum Technologies, Lewes, UK), and images of the layers were taken using a scanning electron microscope (SEM, Tescan Vega3, Brno, Czech Republic) at an accelerated voltage of 20 kV. The images were assessed using ImageJ software (NIH, Bethesda, Rockville, MD, USA).

### 2.4. Fourier-Transform Infrared Spectroscopy

To determine if the prepared samples were actually crosslinked, a measurement of the Fourier-transform infrared spectrometry (FTIR) was performed. FTIR was measured on the fibrous layers before and after crosslinking, using a Nicolet iZ10 instrument (Thermo Fisher, Waltham, MA, USA) and an ATR diamond crystal.

### 2.5. Strength of the Nanofibrous Layers

The strength of the fiber layers before and after crosslinking was measured using a LabTest 6.031 device (Labortech instrument, Opava, Czech Republic) applying a head with a range of up to 150 N, a loading speed of 100 mm/s and a clamped length of 50 mm. Five 100 × 50 mm samples were tested from each set.

### 2.6. Sterilization

The materials were sterilized in an Anprolene AN-74i (Andersen Products, Haw River, NC, USA) sterilizer using ethylene oxide at 37 °C for 12 h. The materials were vented at room temperature for one week following sterilization.

### 2.7. Cell Cultivation

3T3-SA fibroblasts (ATCC, Manassas, VA, USA) cultured in Dulbecco’s modified eagle medium (DMEM, Biosera, Prague, Czech Republic) enriched with 10% fetal bovine serum (Biosera, Prague, Czech Republic), 1% glutamine (Biosera, Prague, Czech Republic) and 1% antibiotics—Pen/Strep Amphotericin B (Lonza Biotec, Kourim, Czech Republic) were selected for the experiment because of their essential support in wound healing [32]. The cells were incubated in an incubator at 5% CO_2_ and 37 °C.

### 2.8. Cytotoxicity

According to the ISO 10993-5:2009 standard, a material is considered to be cytotoxic if the viability of cells incubated with an extract from the material evinces values of lower than 70% of the cell viability of the negative control. [33] Cells from passage 9 at a concentration of 1 × 10^4^ per well were seeded at the bottom of a 96-well microtiter plate and cultured for 24 h. A total of 9 wells of cells were seeded for the positive control (PC), the negative control (NC) and the tested material. A total of 3 sets of samples were prepared from the tested material. The materials were then incubated with the complete medium (DMEM) for 24 h.

The culture medium was then changed to extracts (100 μL per well) of the test materials followed by 0.1% triton in the DMEM for the PC and the pure complete medium (DMEM) for the NC. The cells were further incubated for 24 h, following which their viability was determined via spectrophotometric analysis using the MTT assay. Measurements were then taken using a spectrophotometer (TECAN, Spark, Mannedorf, Switzerland) at 570 nm and 650 nm (the reference values).

## 3. Results

### 3.1. Spinning of the Solution

The optimal process parameters for the production of fibrous layers from the gelatin and alginate solutions were sought during the testing stage; however, it was subsequently determined that neither of the prepared solutions were spinnable.

Due to the inability to produce fibrous layers solely from the gelatin or alginate, they were mixed with PVA, and further experiments were conducted in order to determine the optimal concentration of the various components. Gelatin at concentrations of 8, 10 and 12 wt% of the total amount was then added to the prepared PVA solutions. Alginate was also added to the PVA at concentrations of 2, 4 and 6 wt% of the total amount. Following spinnability testing, solutions composed of 8 wt% of PVA with 8 wt% of gelatin and 8 wt% of PVA with 4 wt% of alginate were finally selected for spinning. The solutions were spun under the conditions listed in Table 1.

### 3.2. Fiber Morphology and Diameter Analysis

**Figure 1:**
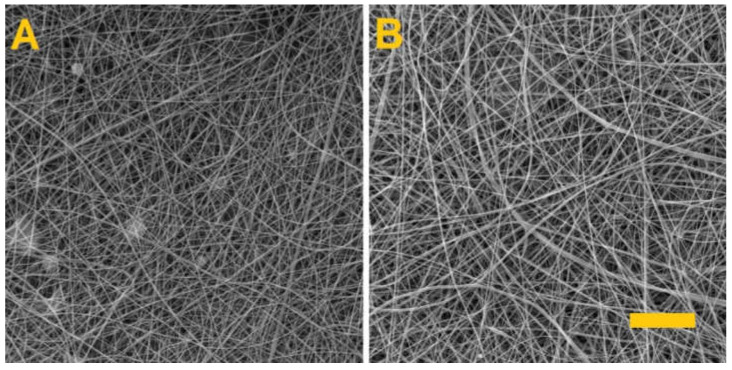
Morphology of the produced fibrous layers. (**A**) PVA/gelatin, (**B**) PVA/alginate. Scale 10 µm.

The fiber layers produced were examined using a SEM. Figure 1 provides SEM images of the PVA/gelatin and PVA/alginate layers and Table 2 lists the parameters of the produced fiber layers.

Whereas the spinning of the PVA/alginate resulted in a homogeneous fibrous layer with no defects, the PVA/gelatin fibrous layer had a fibrous structure with the presence of minor droplet defects that caused the fibrous layer to adhere to the underlying spunbond layer and the tearing of the layer during subsequent handling. In both cases, however, there is a shift in productivity, as fiber layers for the preparation of hydrogel fibers are currently only produced in small quantities [34,35,36].

### 3.3. Fiber Layer Crosslinking

**Figure 2:**
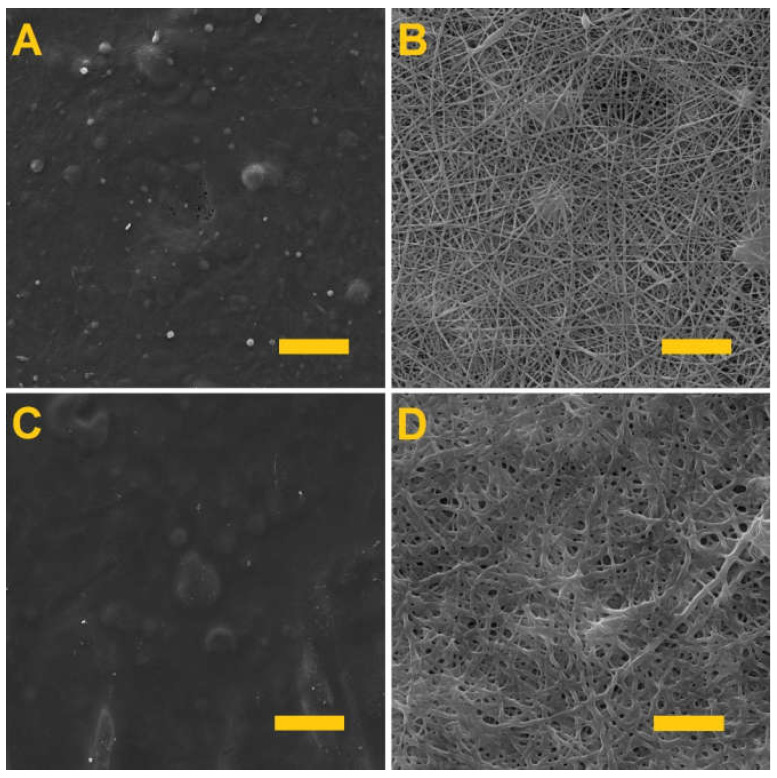
The fiber layers following crosslinking. (**A**) PVA/gelatin after 24 h of crosslinking; scale 50 µm. (**B**) PVA/gelatin after 4 h of crosslinking; scale 10 µm. (**C**) PVA/alginate after 24 h of crosslinking; scale 50 µm. (**D**) PVA/alginate after 4 h of crosslinking; scale 10 µm.

Testing was conducted aimed at determining the optimal crosslinking time that would allow for the crosslinking of the gelling component, i.e., the gelatin and the alginate, without the crosslinking of the PVA. Figure 2 shows the PVA/gelatin layers (A and B) and the PVA/alginate layers (C and D) following differing crosslinking times.

It is clear from Figure 2 that the longer fiber layer crosslinking time led to the formation of films in both cases. Thus, in order to preserve the fibrous structures of the materials, they were both crosslinked for 4 h. This is in contrast to previous publications [37,38], where alginate was crosslinked for 24 h. In the case of nanofibers, therefore, a shorter crosslinking time is sufficient.

### 3.4. Wetting of the Crosslinked Fiber Layers

**Figure 3:**
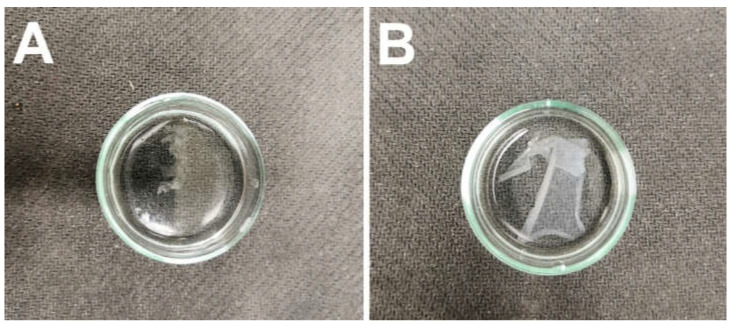
The fiber layers following soaking in distilled water. (**A**) The PVA/gelatin fiber layer. (**B**) The PVA/alginate fiber layer.

Following crosslinking, the fiber layers were subjected to wetting in distilled water, as illustrated in Figure 3. Although testing revealed that the PVA/gelatin fibrous layer did not dissolve during soaking in water, due to its adhesion to the substrate material and subsequent tearing during handling, it was decided that this fibrous layer would be excluded from further testing.

The PVA in the PVA/alginate fibrous layer gradually dissolved upon contact with water accompanied by the swelling of the alginate to form a hydrogel from the produced alginate fibers. Hence, the hydrogel fibers were prepared via this wetting process.

**Figure 4:**
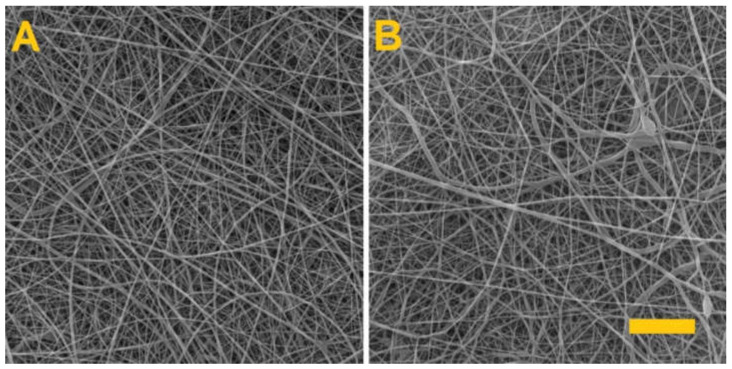
The PVA fiber layer. (**A**) Prior to crosslinking. (**B**) After 4 h of exposure to the glutaraldehyde vapor. Scale 10 µm.

A fibrous layer of 8% PVA was subsequently prepared in order to verify that only the PVA in the PVA/alginate fibrous layer had dissolved. The produced fibrous layer was again crosslinked for 4 h with glutaraldehyde. Figure 4 shows the PVA fiber layers prior to crosslinking and after 4 h of exposure to the glutaraldehyde vapor.

After 4 h of glutaraldehyde vapor exposure, the PVA fibrous layer was placed in distilled water, whereupon it was observed to completely dissolve. Thus, the experiment confirmed that the alginate had crosslinked during the crosslinking of the PVA/alginate fibrous layer.

### 3.5. FTIR

**Figure 5:**
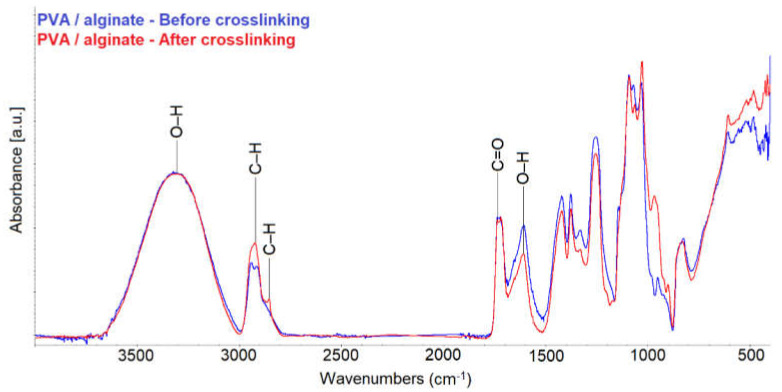
FTIR spectra of the PVA/alginate fiber layers before and after crosslinking.

FTIR measurements was performed to confirm that crosslinking occurred in the PVA/alginate sample. Figure 5 shows the spectra of the PVA/alginate fiber layers before and after crosslinking.

The measured spectra show that there was a change in the structure of the PVA/alginate layer after crosslinking. Due to crosslinking, there was no change in the absorbance for the O–H group in the peak at 3360 cm^−1^. However, a change of absorbance in C–H is observed for CH_3_ (peaks at 2870 and 2930 cm^−1^) and for CH_2_– (peaks at 2850 and 2920 cm^−1^). Another change occurred in the O–H group peak at 1630 cm^−1^. From the measured absorbances it can be deduced that glutaraldehyde was bound between the O–H groups of the alginate, as reported in the publications [39,40]. The measured spectra also show a change in the vibration of the skeleton. Thus, cross-linking occurred due to glutaraldehyde vapor.

### 3.6. Strength of the Fibrous Layers

**Figure 6:**
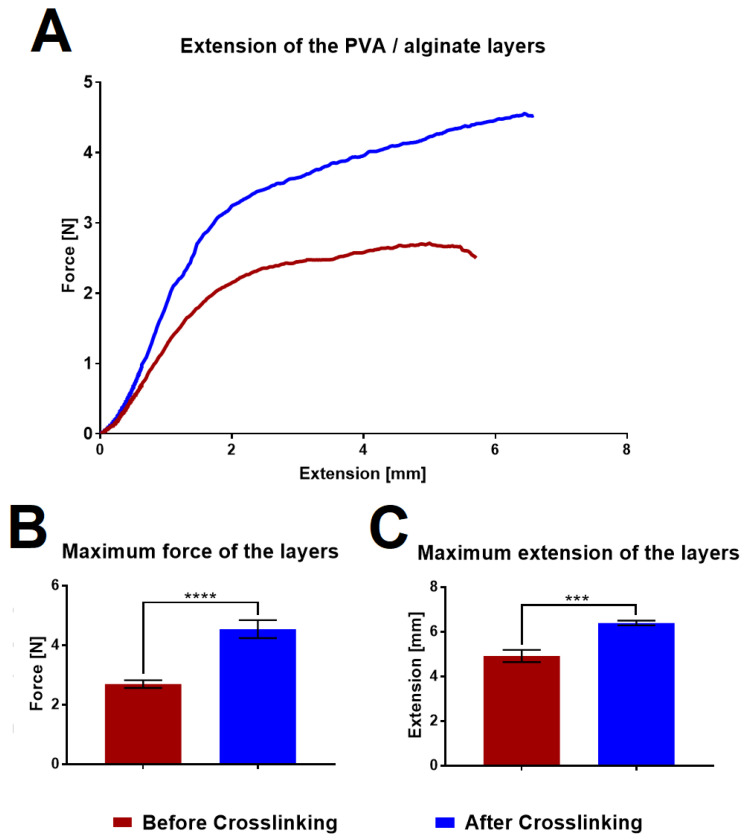
Mechanical testing of the PVA/alginate layers. (**A**) Results of the tensile testing of the layers before (burgundy color) and after (blue color) crosslinking. (**B**) Maximum layer strength before and after crosslinking. (**C**) Maximum elongation of the layers before and after crosslinking. 95% confidence interval (CI); *** *p* < 0.0006, **** *p* < 0.0001.

The strength of the PVA/alginate fibrous layers was measured using a Labortech device in order to determine the differences in their properties before and after crosslinking. The graph in Figure 6 shows the tensile curves and a comparison of the maximum strength and maximum elongation that the layers are able to achieve before they break.

The graphs illustrate that the fiber layers after crosslinking significantly evinced both higher strengths and elongation, thus indicating that the fibrous layers will be easier to handle in the case of application. If we compare the values with other fiber wound dressing [41,42], the tested layers achieve lower strength, but at the same time achieve higher ductility. An increase in strength could be achieved by increasing the area weight of the sample.

### 3.7. Cytotoxicity

**Figure 7:**
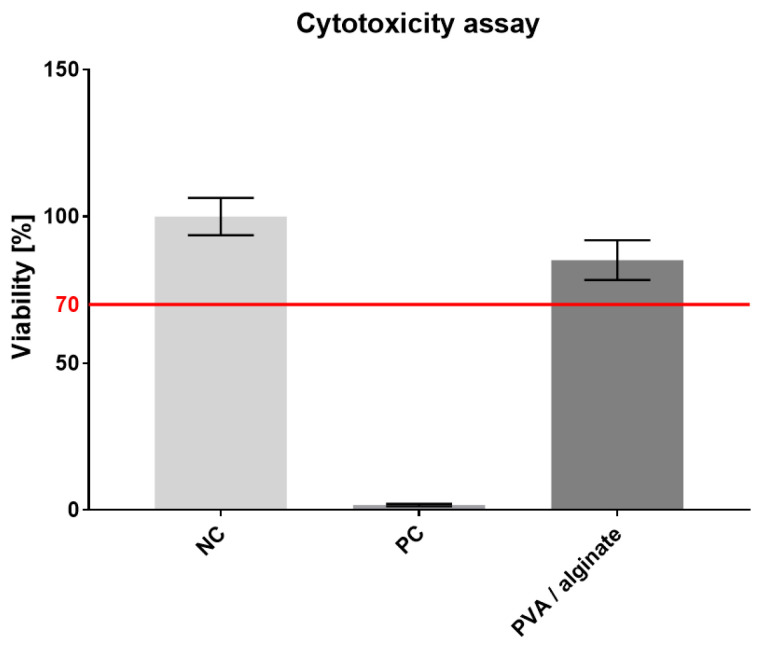
PVA/alginate fibrous layer cytotoxicity assay, 95% CI.

The resulting crosslinked PVA/alginate fiber layers were tested for cytotoxicity. A microscopic inspection of the cell morphology was performed on the third day, followed by an MTT assay aimed at determining the metabolic activity of the cells. The results of spectrophotometric absorbance measurements of NC, PC and tested material are shown in the graph in Figure 7.

The metabolic test proved that the prepared material was not cytotoxic, which is important particularly in terms of the crosslinking applied and subsequent applications for its use for skin cover purposes. However, glutaraldehyde has a slight effect on the resulting cell viability. In comparison with other PVA/alginate fibers which were crosslinked by thermal treatment [43], our fibers have slightly lower cell viability. Cell viability from the PVA/alginate extract reached 85%.

### 3.8. Production of a Modified Fiber Layer

The experiment also included the production of a modified fiber layer. Albumin (Sigma-Aldrich) was added to the PVA/alginate solution (8% PVA with 4% alginate) and the maximum concentration thereof was investigated for spinning purposes. Albumin was selected for the modification of the fibrous layer due to its potential use in the treatment of skin burns. The experiment involved the addition of 2, 4, 6, 8 and 10 wt% (total weight of the solution) of albumin to the solution. Since the experimental research previously determined that the maximum concentration of alginate that allows for spinning and the formation of a homogeneous fibrous layer is 8 wt%, it was decided to produce a PVA/alginate fibrous layer with an albumin content also of 8%. The production parameters of the modified fiber layer are listed in Table 3.

**Figure 8:**
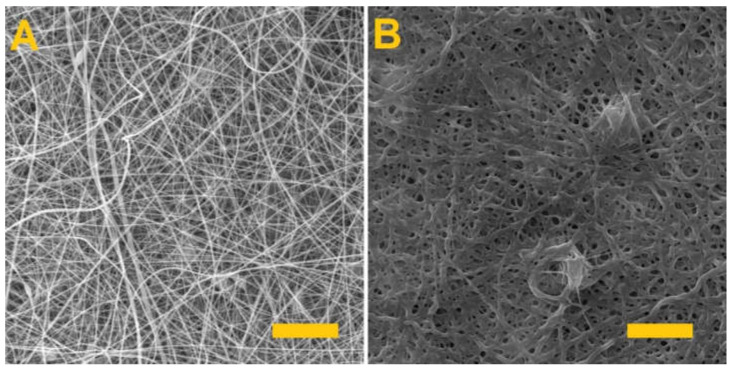
The PVA/alginate fiber layer with albumin. (**A**) Fiber layer before crosslinking; scale 50 µm. (**B**) Fiber layer after 4 h of crosslinking; scale 10 µm.

The resulting modified fiber layer was again crosslinked with glutaraldehyde for 4 h. Figure 8 illustrates the PVA/alginate/albumin layer before and after crosslinking.

As can be seen from the images, homogeneous fibrous layers of PVA/alginate/albumin were made. Crosslinking after 4 h led to a change in structure, but the fibrous structure of the produced layer was still preserved.

### 3.9. Cytotoxicity of the Modified Fibrous Layer

**Figure 9:**
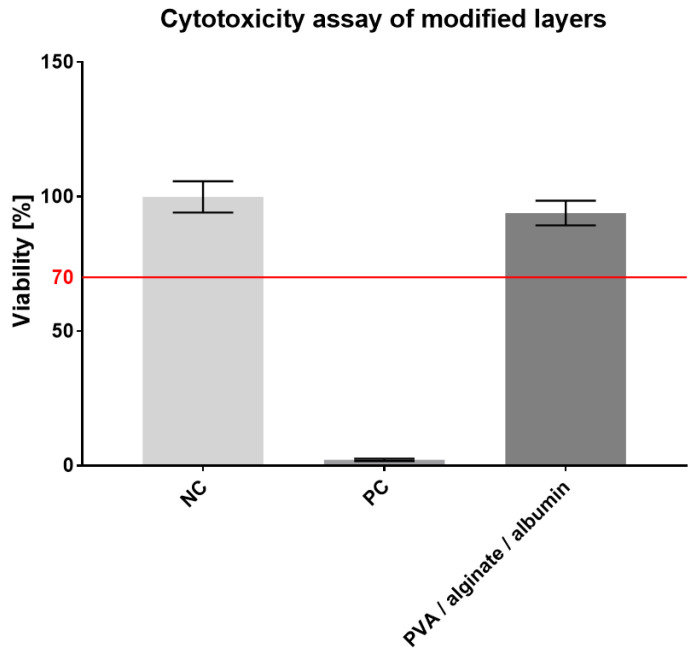
Cytotoxicity assay of the modified PVA/alginate/albumin fiber layer, 95% CI.

As with the original layer, the modified fibrous layer was subsequently tested for cytotoxicity. In this case, a no. 13 cell passage of 3T3 fibroblasts was seeded into microtiter plate wells at the same concentration as the original layers, i.e., 10^4^ cells/100 µL per well in the culture medium. An MTT test was again performed on the third day; the resulting values are shown in the graph in Figure 9.

The metabolic test results proved that the modified material was not cytotoxic and that the added albumin led to a higher cell viability (94%) than the unmodified PVA/alginate layers did (85%). The modified material can, therefore, be considered for use as a skin cover.

## 4. Conclusions

The experiment involved the determination of suitable materials for the fabrication of hydrogel fibers. Since it was discovered that aqueous solutions of gelatin and alginate could not be spun on the Nanospider device, they were subsequently combined with PVA. The various components of the PVA/gelatin and PVA/alginate blends and the electrospinning process parameters were then optimized. During the study, a semi-industrial production of fiber layers was performed. This is an improvement over previous studies that produced only a small amount of fibers [34,35,36]. The fiber layers produced from the PVA/gelatin and PVA/alginate blends were investigated so as to determine the time required to crosslink the gelling component, i.e., the gelatin and the alginate, which resulted in the selection of 4 h of crosslinking. As found in this and other studies [37,38], a longer time in glutaraldehyde vapor leads to crosslinking of PVA. In our study, a longer crosslinking time led to the growth of the PVA and the formation of a polymer film. Since it was later found that the PVA/gelatin layer tore during handling, it was excluded from further consideration. Thus, the PVA/alginate layer was chosen for further testing involving the investigation of the mechanical properties of the material, which revealed that the crosslinking of the PVA/alginate layer led to enhanced strength and ductility, and thus, to improved handling properties during application. The cytotoxicity testing concluded that the fibrous layers were non-toxic, which is important particularly in terms of the crosslinking applied to the fibrous layers. The experiment also involved the fabrication of a modified fibrous layer with the addition of albumin. The cytotoxicity test proved that this layer was also non-toxic. Thus, the results of the study indicate that this layer can be used in skin covers for the treatment of burns. However, this is an initial study and there is a need to investigate the properties of the layers produced, such as monitoring albumin release.

## Figures and Tables

**Table 1:** Process parameters of the spinning of the selected solutions.

	PVA/Gelatin	PVA/Alginate
Distance between the electrodes [mm]	160	145
Voltage of the collector [kV]	−10	−10
Voltage of the electrode [kV]	50	60
Spunbond towing speed [mm/min]	5	
Temperature [°C]	22	
Relative humidity [%]	45	

**Table 2:** Parameters of the produced fiber layers.

	PVA/Gelatin	PVA/Alginate
Fiber diameter [µm]	0.249 ± 0.059	0.368 ± 0.102
Area weight [g/m^2^]	2.01 ± 0.08	1.9 ± 0.09

**Table 3:** Production parameters of the modified fiber layer.

	PVA/Alginate/Albumin
Distance between the electrodes [mm]	160
Voltage of the collector [kV]	−10
Voltage of the electrode [kV]	50
Spunbond towing speed [mm/min]	5
Temperature [°C]	22
Relative humidity [%]	45

## Data Availability

The data presented in this study are available on request from the corresponding author.
